# Review on Carbon/Polyaniline Hybrids: Design and Synthesis for Supercapacitor

**DOI:** 10.3390/molecules24122263

**Published:** 2019-06-18

**Authors:** Xiaoning Wang, Dan Wu, Xinhui Song, Wei Du, Xiangjin Zhao, Dongmei Zhang

**Affiliations:** 1 School of Environment and Material Engineering, Yantai University, Yantai 264005, China; yantaiwxn@163.com (X.W.); 18809498404@163.com (D.W.); Songxinhui0413@163.com (X.S.); 2 College of Nuclear Equipment and Nuclear Engineering, Yantai University, Yantai 264005, China; 3 Shandong Institute for Food and Drug Control, Jinan 250101, China; zhangdm1000@163.com

**Keywords:** polyaniline, carbon material, composites, supercapacitors

## Abstract

Polyaniline has been widely used in high-performance pseudocapacitors, due to its low cost, easy synthesis, and high theoretical specific capacitance. However, the poor mechanical properties of polyaniline restrict its further development. Compared with polyaniline, functionalized carbon materials have excellent physical and chemical properties, such as porous structures, excellent specific surface area, good conductivity, and accessibility to active sites. However, it should not be neglected that the specific capacity of carbon materials is usually unsatisfactory. There is an effective strategy to combine carbon materials with polyaniline by a hybridization approach to achieve a positive synergistic effect. After that, the energy storage performance of carbon/polyaniline hybridization material has been significantly improved, making it a promising and important electrode material for supercapacitors. To date, significant progress has been made in the synthesis of various carbon/polyaniline binary composite electrode materials. In this review, the corresponding properties and applications of polyaniline and carbon hybrid materials in the energy storage field are briefly reviewed. According to the classification of different types of functionalized carbon materials, this article focuses on the recent progress in carbon/polyaniline hybrid materials, and further analyzes their corresponding properties to provide guidance for the design, synthesis, and component optimization for high-performance supercapacitors.

## 1. Introduction

**Figure 1:**
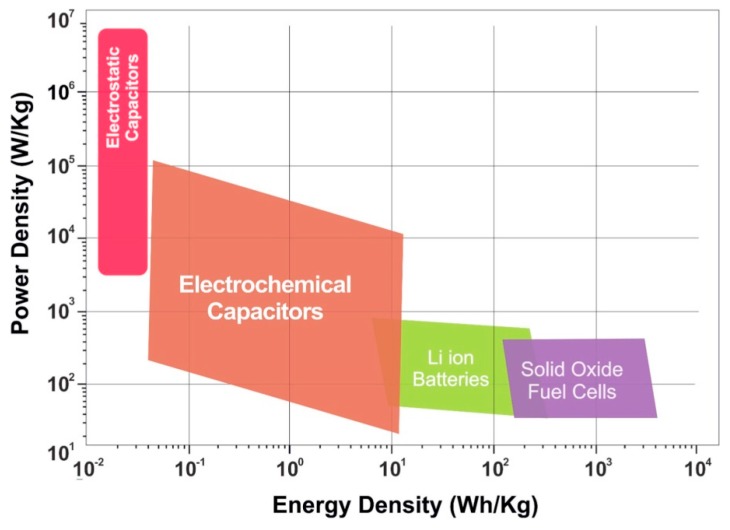
Ragone plots for various electrochemical energy storage systems. Reproduced with permission from [7], copyright New York Ny: American Institute of Physics, 2017.

Nowadays, the prosperity of the world economy has been heavily dependent on the exploitation and utilization of fossil fuel resources, such as coal, natural gas, and petroleum [1]. The annual demand for these resources is still showing a rapid increase. The depletion of fossil fuel resources has caused a series of economic and societal problems with the increasing exploitation of fossil fuel resources [2]. In the past decade, the extensive development and application of various emerging energy sources, such as hydro energy, biomass energy, wind energy, and tidal energy, have greatly alleviated the serious problems existing in the fields of energy and environment [3]. However, this renewable energy source is sufficiently stored and utilized in the form of being finally converted into electric energy. Therefore, there is an urgent need to develop a stable electrochemical energy storage system (such as a supercapacitor, an ion battery, and a fuel cell) to achieve efficient storage of these energy sources [4,5]. Among them, supercapacitors, also called electrochemical capacitors, are novel green energy storage devices based on interface ion-based adsorption and desorption (an electrochemical double layer capacitor (EDLC)) or rapid redox reactions (a pseudo-capacitor (PC)) to store energy [6]. Compared with fuel cells and ion batteries, supercapacitors have the advantages of high output power density, long service life, low cost, and a fast charging and discharging process, which make them one of the most promising electrochemical storage systems, as is evident from the Ragon plots shown in Figure 1 [7].

The energy storage performance of supercapacitors is determined by a variety of factors, such as the physical and chemical properties of the electrode active materials, the choice of electrolyte type (aqueous, organic, ionic liquid, solid- or quasi-solid-state, and redox-active electrolytes), the assembly mode of the electrode, and the choice of working potential window [8]. In particular, the inherent electrochemical energy storage capacity of a supercapacitor is highly dependent on the type of electrode material and the bonding mode of the molecular-scale or physical mixing. So far, a number of research groups have been working on advanced electrode materials for supercapacitors that have perfect material properties to facilitate electron transport and ion diffusion. Carbon materials and polyaniline (PANI) materials, as the most representative materials of EDLCs and pseudocapacitors, respectively, show their unique advantages in two types of energy storage fields.

Carbon is widely present in the atmosphere, crust, and organisms in various forms and plays a vital role in the normal functioning of the ecosystem [9]. The development of the human economy is closely related to the struggle to extract and use related energy from carbon materials in the earth’s crust. Carbonaceous materials are formed by carbon atoms through different bonding methods. In particular, functional carbon materials have attracted extensive research in the field of supercapacitors owing to their low cost, good physical and chemical stability, diverse porous structures, excellent specific surface area, good ionic conductivity, and accessibility to active sites. In general, carbon materials are used in EDLCs due to their electric double layer principle without any other synthesis or doping processes [10]. The capacitance of the EDLCs is induced by the accumulation of electrostatic charges on the electrode interface. The accumulation of charge is formed by rapid physical reversible adsorption/desorption between the electrode surface and the electrolyte without any chemical reaction. The charge storage space of EDLC is concentrated in electrodes, the diffusive layer in electrolytes, and the Helmholtz layer. The energy storage principle determines that EDLCs can provide extremely high power density, charge, and discharge rates and extremely high two-pass efficiency, safety, and excellent cycle stability. However, the specific capacitance and energy density of the pure EDLC materials (100~300 F g^−1^, 5~7 Wh kg^−1^) are determined and limited by their ion accessible surface area. Therefore, excellent EDLC materials often require a high specific surface area, large porosity, and proper pore distribution. Fortunately, the discovery of a variety of new functional carbon nanomaterials has created favorable conditions for the improvement of energy density with the development of science and technology. These functional carbon nanomaterials include: Activated carbon (AC), carbon nanotubes (CNTs), carbon fibers (CFs), and graphene (including graphene oxide (GO) and reduced graphene oxide (rGO)). It is worth noting that there is a huge gap between the specific capacitance and energy density of EDLCs and PCs due to the limitation of the electrostatic surface charging mechanism. It is well known that carbon materials are easily combined with metal oxides and polymer conductive polymers, resulting in anisotropic and synergistic multi-element new electrode materials. Therefore, these new and diverse carbon-based composites have a promising application potential in the electrochemical field of batteries, supercapacitors, and electrocatalysts.

PANI is a common conductive polymer and one of the most representative PC materials. Since it was first synthesized in 1886, PANI has become the most promising conductive polymer, due to its easy synthesis, low cost, high theoretical specific capacitance (~1200 F g^−1^), good conductivity, special doping mechanism, excellent wave absorption, and electrochemical performance [11]. PANI is polymerized by oxidation of an aniline monomer through an electrochemical or chemical route [12]. Aniline is one of the most important amines, and it is mainly used in the manufacture of dyes, drugs, resins, and as a rubber vulcanization accelerator. Therefore, PANI is the cheapest and the most thermally stable of intrinsically conductive polymers [13]. In view of the above advantages, the development of PANI as a typical PC material has achieved outstanding research output in the field of supercapacitors. For PCs, the charge storage mechanism can be divided into reversible redox reactions, underpotential deposition, and intercalation pseudocapacitance. In general, PCs can exhibit high rate performance and store more charge than EDLCs, which stems from the fact that reactions in the surface and bulk of the electrode material are not limited by solid state diffusion. In addition, the pseudocapacitive materials also depend on the morphology or intrinsic properties, and it can be intrinsic to a material or extrinsic. The intrinsic pseudocapacitive material exhibits the characteristics of capacitive charge storage and is suitable for various particle sizes and forms [14,15,16]. Among them, PANI has a high capacitance value, due to its multiple redox states, and can also cause a large total surface charge potential during charge and discharge when transferring from one oxidation state to another. However, PANI may swell, shrink, or degrade during long-term charging/discharging, due to structural damage of the main chain, resulting in poor conductivity and stability [13]. An effective solution is to design ordered PANI nanorods and nanoparticles [17]. Ordered PANI nanostructures have high specific surface area, good cycle stability, high energy storage capacity, and excellent rate performance, compared to randomly connected geometries [18]. In addition, the synergistic composite method between PANI and other active materials has been widely studied, which can improve the conductivity of metal oxides, the poor stability of PANI, and the specific capacitance of carbon materials, thereby achieving high electrochemical performance. Carbon materials are one of the candidate materials for achieving these properties in all composites with PANI. A variety of functional carbon materials are incorporated into the PANI nanostructured materials to significantly enhance their electrical and mechanical stability. Therefore, the hybrid electrode material of the nanostructure-based PANI and carbon material can not only provide excellent conductivity and stability from the carbon material, but also exhibit a synergistic effect from the high pseudo-capacitance of PANI.

**Figure 2:**
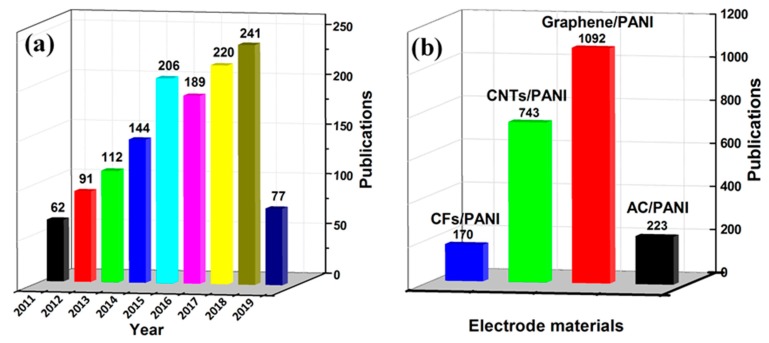
Statistical survey on the research activities toward supercapacitors: (**a**) Total number of reported literature related to carbon/carbon materials and polyaniline (PANI) composites for supercapacitors from 2011 to 2019; (**b**) literature publication statistics for different carbon materials/PANI composites. Source of the data: Web of Science; search time: 20 May 2019.

This review focuses on providing cutting-edge insights by summarizing the available knowledge of supercapacitors for different functional carbon/PANI composites in recent years. The papers published in recent years based on carbon/PANI composites in the field of supercapacitors are shown in Figure 2. As shown in Figure 2a, the number of papers in the field of carbon/PANI composites is increasing year by year, which indicates that its research work is still the current hotspot for supercapacitor electrode materials. Figure 2b shows the annual research hotspot of new electrode materials, which clearly indicates that carbon/PANI research has focused on graphene in recent years. Finally, this review also presents the conclusions and future prospects for the development of high-performance supercapacitors through the use of novel functional carbon/PANI composites.

## 2. Properties and Applications of Carbon/Polyaniline Hybrids for Advanced Supercapacitors

### 2.1. Activated Carbon/Polyaniline Hybrids

The AC as a carbonaceous material is predominantly amorphous, and its composition contains 80% or more carbon. However, the inherent poor conductivity of amorphous carbon is an inevitable bottleneck for its development in the field of energy storage. In order to improve the applicability of ACs, researchers generally use high-temperature local graphitization and artificial graphite coating to improve the conductivity of amorphous carbon [5,17]. Generally, AC has a large specific surface area, so that it has good adsorption capacity for solid particles in gases and liquids [19]. In addition, AC materials have been widely used in the fields of energy storage, the chemical industry, environmental protection, metallurgy, and military chemical protection, due to their stable chemical properties, good sustainability, high mechanical strength, and excellent acid and alkali resistance. Since 1957, AC has been used in the field of energy storage, and its related research has been rapidly developed in recent years [20]. Based on extensive research already carried out in the field of preparation and characterization of AC, it has been established that the physical and chemical properties of AC depend on the choice of precursor matrix and type of activation [21]. Most commercial ACs are produced by pyrolysis of fossil fuel-based precursors at elevated temperatures and pressures, which makes them expensive, non-renewable, and environmentally unfriendly [22]. For biomass materials, readily degradable components, such as hemicellulose, cellulose, lignin, and tannin form low cost, green and structurally-stable three-dimensional channels and pore structures by chemical, thermochemical, or biological treatment [23,24]. In the field of supercapacitor applications, the inherent structure of the biomass precursor has an important influence on the microstructure and porosity of the carbonized product. Therefore, carbon nanomaterials having various biomimetic microstructures and morphology can be obtained by skillfully selecting biomass precursors. For excellent carbon electrode materials, AC with multi-level hierarchical pore structure provides the necessary ion buffer reservoir, fast ion transport channel, and ion storage site for electrode materials [25]. To sum up, biomass-based AC materials are widely used in EDLC electrode materials, due to their advantages of heteroatom self-doping, low cost, environmental protection, and renewable, unique pore structure, large pore capacity, excellent chemical stability, and electrical conductivity.

**Figure 3:**
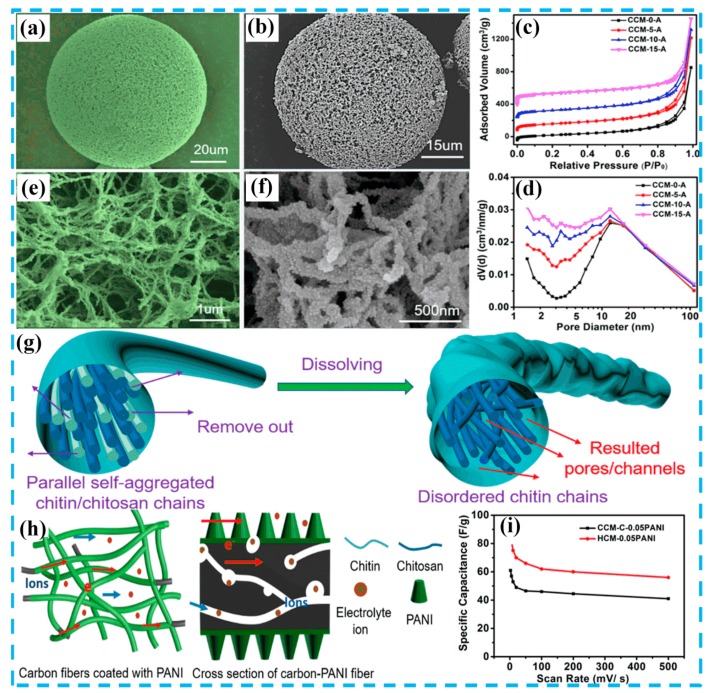
(**a**,**e**) SEM images of chitin/chitosan microspheres (CCMs); (**b**,**f**) SEM images of CCMs/PANI; (**c**) nitrogen adsorption and desorption isotherms; (**d**) Barrett−Joyner−Halendar (BJH) pore size distribution samples; (**g**) graphical illustrations for the change of nanofibers during the acid treatment process; (**h**) graphical illustrations of the formation process for CCM/PANI; (**i**) specific capacitance based on the total mass of the two electrodes at different scan rates. Reproduced with permission from Reference [37], copyright American Chemical Society, 2018.

To date, literatures have demonstrated that composite materials based on PANI and AC can provide a synergistic combination of high porosity from carbon and high pseudo capacitance from PANI [26,27,28,29,30,31,32,33,34,35]. Inevitably, the interface resistance in nanostructures is a bottleneck in the development of conductive polymer materials [36]. Many researchers have wisely combined AC materials with PANI to overcome these disadvantages, making them the best candidates for supercapacitors. As shown in Figure 3, Gao et al. [37] prepared hierarchically porous carbon microspheres (HCMs) by pyrolysis of chitin microspheres (which were synthesized from a chitin and chitosan blend solution), in which chitosan was used as the formation agent for the nanopore/nano-channel to construct the microspheres. The internal structure of the HCM presented a unique hierarchically porous structure with a specific surface area of 1450 m^2^ g^−1^. Subsequently, PANI nanorods were synthesized on the surface of the HCM. The assembled symmetric supercapacitor exhibited significant rate performancei and cycle stability (capacitance retention: 90.6%) after 10,000 cycles, indicating that the HCM has good applicability for supercapacitors. Another work on the synthesis of carbon spheres was done by Lyu et al. [38], using yeast as a carbon source and structural template. In this work, yeast-derived N-doped carbon microspheres (YCs)/PANI hybrids were synthesized by in situ polymerization. The prepared YCs have good electrical conductivity and many active sites, which can alleviate the structural damage caused by PANI in the charge-discharge reaction. The YC/PANI composite exhibited a high specific capacitance (500 F g^−1^ at a current density of 1 A g^−1^) in a three-electrode system.

**Figure 4:**
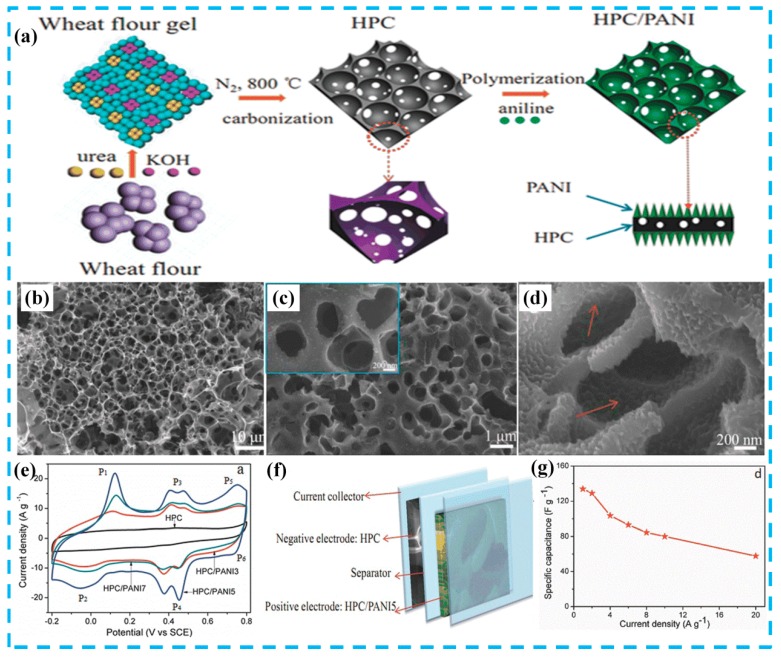
(**a**) Schematic illustration for the preparation of hierarchically porous nitrogen doped carbon (HPC)/PANI composites; (**b**) SEM image of HPC; (**c**,**d**) SEM images of HPC/PANI hybrids; (**e**) Cyclic voltammetry (CV) curves at a scan rate of 50 mV s^−1^ of HPC and HPC/PANI electrodes measured in a three-electrode system; (**f**) schematic diagram of the structure of the asymmetrical supercapacitor (ASC) device based on HPC and HPC/PANI electrodes; (**g**) specific capacitance of HPC/PANI//HPC based on the total mass of the two electrodes versus current density. Reproduced with permission from Reference [17], copyright Wiley, 2016.

In addition, AC has another type of structural form: Hierarchically porous carbon. For example, Yu et al. [17] synthesized a hierarchically porous nitrogen doped carbon (HPC)/PANI nanocomposite by in situ polymerization. As shown in Figure 4a, wheat flour, as a carbon precursor, was further carbonized after treatment with urea and alkali to prepare three dimensional interconnected honeycomb HPCs. The prepared HPC exhibited a high surface area (1294 m^2^ g^−1^) and a specific capacitance of 383 F g^−1^. Finally, the asymmetric supercapacitor was successfully assembled in Figure 4f. The device exhibited high specific capacitance (134 F g^−1^), high energy density (60.3 Wh kg^−1^) and power density (18 kW kg^−1^), and excellent cyclic stability (91.6% capacitance retention after 5000 cycles) in neutral electrolytes. For comparison, this review summarizes the preparation and electrochemical performance of some typical AC/PANI electrode materials, as shown in Table 1 (In Table 1, Table 2 and Table 3, the specific capacitance and capacitance retention are characterized in a three-electrode system. Power density and energy density are characterized in a two-electrode system).

### 2.2. Graphene or Graphene Oxide/Polyaniline Hybrids

**Figure 5:**
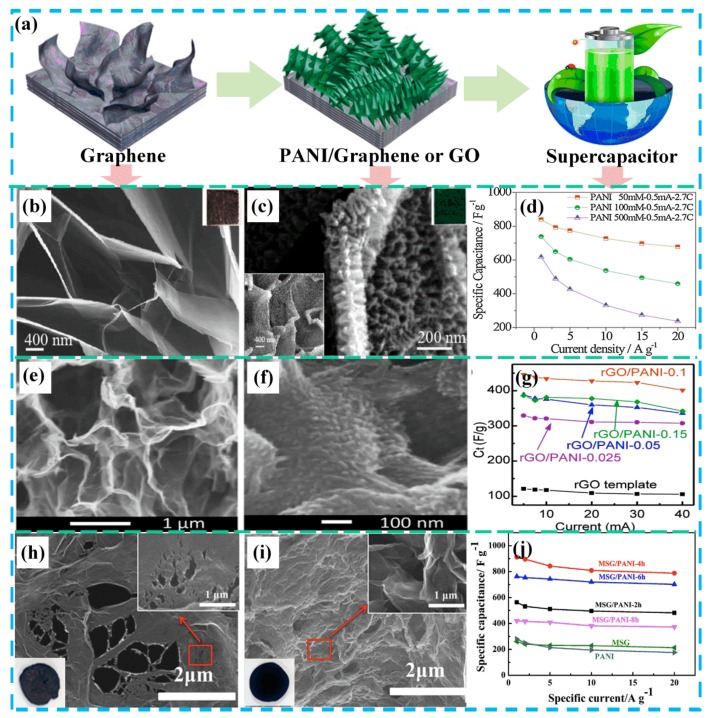
Schematic of a reasonable design for graphene or GO/PANI-based supercapacitors: (**a**) Schematic of the manufacturing process of PANI attached to graphene or graphene oxide (GO); (**b**) SEM images and digital photos of as-prepared exfoliated graphite foil; (**c**) SEM images and digital photos of exfoliated graphite foil/PANI; (**d**) the specific capacitance of exfoliated graphite foil/PANI at different current densities. Reproduced with permission from Reference [63], copyright Elsevier, 2017. (**e**) Cross-section SEM images of the reduced graphene oxide (rGO) template; (**f**) cross-section SEM images of rGO/PANI clearly showing the formation of PANI on the rGO sheets; (**g**) the specific capacitance of PANI/rGO/PANI electrodes at various charge/discharge currents. Reproduced with permission from Reference [64], copyright Elsevier, 2017. (**h**) SEM images and digital photos of the multi-growth site graphene; (**i**) SEM images and digital photos of the multi-growth site graphene/PANI composite; (**j**) T = the specific capacitance of graphene, PANI, and the multi-growth site graphene/PANI composite. Reproduced with permission from Reference [65], copyright Elsevier, 2018.

Graphene, a fascinating and multifunctional material, was discovered by Novoselov and Geim using micromechanical exfoliation, in 2004 [39]. Graphene is a two-dimensional carbon nanomaterial with a honeycomb lattice shape, consisting of carbon atoms in the sp^2^ hybrid orbital, and it is also the thinnest two-dimensional material discovered so far [40]. This unique crystal lattice endows graphene with high breaking strength (~42 N m^−1^), high Young’s modulus (~1 TPa), strong physicochemical durability, and high electron mobility (~2.5 × 10^5^ cm^2^ V^−1^ s^−1^). Therefore, it has important application prospects in the fields of sensing, energy storage, medicine, semiconductors, and biomaterials, and it is even considered to be a revolutionary functional material [41]. Initially, graphene was not considered a good candidate material for universal adoption because it is too expensive to manufacture and difficult to make scalable [42]. In recent years, several new technologies for graphene production have been developed, which replace the traditional micromechanical exfoliation method, such as the redox method, the SiC epitaxy growth method, and the chemical vapor deposition (CVD) method [43,44]. These synthetic methods give graphene excellent structure and electrochemical properties, such as high specific surface area (~2630 m^2^ g^−1^), high electrical conductivity (~10^6^ S cm^−1^) at room temperature, stable electrochemical properties, and high theoretical specific capacitance (~550 F g^−1^), which make it a promising material for supercapacitors [45]. Recently, a variety of novel work has been published on graphene and PANI composites, showing new or highly improved properties [46,47,48,49,50,51,52,53,54,55,56,57,58,59,60]. The combination of graphene and PANI on the nanometer scale can induce positive synergistic effects, which can fully utilize the stable EDLC of graphene and the high PC of PANI. Specifically, graphene can improve the stability of PANI, maintain a high specific surface area, and significantly increase the electrical conductivity of the material, while PANI can inhibit the occurrence of agglomeration of graphene. It is worth noting that graphene can provide considerable and accessible electron and ion pathways for high-speed energy storage. The mechanism behind it depends on the cell system and the interface binding properties of graphene/PANI. In other words, the interface between the graphene surface and the PANI acts as a path separation and electron/ion transport [61,62]. Therefore, in the field of supercapacitors, the research prospects of graphene/PANI nanocomposites tend to design reasonable microstructures and construct ideal three-dimensional porous structures, as shown in Figure 5a. The purpose of all research work should be to avoid the expansion and contraction of PANI, improve the weak interfacial interaction between graphene and PANI, and seek to balance the performance and function of graphene.

The synthesis of the graphene electrode materials is mostly carried out by means of a binder-free method, that is, by depositing a conductive polymer on a graphene electrode, which can overcome the disadvantages of large resistance and poor stability of the powder sample. As shown in Figure 5b,c, Ye et al. [63] synthesized an ordered PANI nanowire array on a graphene sheet from a graphite substrate to form a hierarchical nanostructure. In this way, the “dead volume” limitation caused by the PANI stacking was effectively reduced. In addition, the seamless bonding between the ordered PANI nanowire arrays and graphene nanosheets provides a pathway for the rapid transfer of electrons and ions. The presence of graphene reduces the expansion and contraction of PANI, thereby ensuring high cycle stability. The ordered PANI nanowire/graphene sheet exhibited a high capacitance of 3.57 F cm^−2^ (607 F g^−1^ at 1 A g^−1^) and a good capacitance retention rate (80.4% after 10,000 galvanostatic charge-discharge (GCD) cycles), as shown in Figure 5d. In order to construct a three-dimensional GO composite structure, the method of diffusion-driven layer-by-layer assembly is proposed and adopted as a simple and versatile method. This synthetic method provides a simple and feasible method for producing a three-dimensional porous graphene skeleton. As shown in Figure 5e–g, Hong et al. [64] successfully synthesized three-dimensional rGO using the diffusion-driven layer-by-layer technique. Subsequently, the rGO/PANI composite was synthesized by the method of in situ polymerization, using the three-dimensional rGO as a framework. In this structure, the rGO/PANI combination provides a strong porous structure that contributes to the diffusion of electrolyte ions. The rGO/PANI composite exhibited a high specific capacitance (438.8 F g^−1^ at 0.5 A g^−1^) in a three-electrode system. In addition, the PANI nanoparticles synthesized with rGO as a template reached an ultra-high specific capacitance of 763 F g^−1^ and a capacitance retention of 76.5% (2000 GCD cycles).

It is well known that the surface of graphene lacks vacancies and functional groups that are beneficial for energy storage. The specific capacitance and rate performance of graphene/PANI composites can be significantly improved by increasing the active sites of graphene. Zheng et al. [65] developed a method for preparing a modified three-dimensional graphene/PANI hybrid electrode, as shown in Figure 5g,h. Specifically, they introduced carbon vacancy defects and oxygen functional groups into the surface of graphene nanosheets by acidic oxidative chemical treatment (70% HNO_3_/30% H_2_O_2_) to prepare multi-growth sites of graphene. The specific capacitance (Figure 5i) of the prepared multi-growth site graphene/PANI nanocomposites reached 912 F g^−1^ at 1 A g^−1^, much higher than the initial GO/PANI (432 F g^−1^). Its capacitance retention rate reached 86.4% at a high current density of 20 A g^−1^, and its cycle stability reached 89.5% after 10,000 charge and discharge cycles (10 A g^−1^). A number of recent reports on PANI/GO composites are summarized in Table 2. From the above data, it can be concluded that the microstructure and morphology of PANI/GO are designed to effectively improve electrochemical energy storage.

### 2.3. Carbon Nanotubes/Polyaniline Hybrids

CNTs, also known as bucky tubes, are one-dimensional quantum materials with a special structure. The carbon atoms in the CNTs are dominated by sp^2^ hybridization, and the hexagonal mesh structure has a certain degree of curvature, forming a spatial topology. In addition, CNTs also have a certain amount of sp^3^ hybrid bonds, that is, the chemical bonds contained in the CNTs have both sp^2^ and sp^3^ mixed hybrid states [66]. To some extent, CNTs can be considered to be formed by the curling of graphene sheets. Therefore, according to the number of layers of graphene sheets formed, it is also called single-walled CNTs (SWCNTs), double-walled CNTs (DWCNTs), and multi-walled CNTs (MWCNTs) [67]. The P electrons of the carbon atoms form π bonds on the surface of the CNTs, so that the CNTs have excellent physical properties, such as high tensile strength (50~200 GPa), high tensile modulus (640 GPa~1 TPa), good flexibility, good corrosion resistance, high electrical conductivity (1000~2000 S cm^−1^), high elasticity, and excellent thermal stability (>700 °C in air, 2800 °C in vacuum) [68].

In recent years, PANI/CNT composites have attracted considerable attention in the field of energy storage [69,70,71,72,73,74,75,76,77,78]. The synthesis mechanism of CNT and PANI composites is based on electrostatic interaction and π-π stacking between the aromatic structure and the graphite surface. In addition, the interaction between the carboxylic acid/acyl chloride group of the modified CNTs and the amino group of the aniline monomer is also an important factor for the combination of the two phases [79]. In the field of supercapacitors, CNTs have a stretchable elastic skeleton, which ensures an ideal conductive path for rapid charge propagation, and can also effectively alleviate the volume change of the material during charging/discharging of the electrode material, thereby improving the stability of the device. Increasing the dispersion and solubility of CNTs in solvents is another challenging study. To this end, many researchers have attempted to improve the wettability of CNTs in solvents by surface functionalization of hydrophilic groups. Yang et al. [80] synthesized novel polypyrrole-bonded/CNT composites by chemical oxidative polymerization on plasma-activated CNTs. The air-plasma activated CNTs strategy effectively increases the oxygen-containing groups on the surface of the CNTs, improves the wettability and dispersibility of the CNTs, and provides an active site for forming a conjugated structure for the pyrrole nitrogen group. Another effective method to improve the composite properties of PANI/CNTs is to oxidize the CNTs in a concentrated acid or acid mixture to effect surface modification to form hydroxyl groups, carboxylic acids, and other oxygen-containing groups on the surface. These surface-functional groups allow the CNTs to be uniformly dispersed in the reactants and also enhance the interfacial affinity between the PANI and CNTs, further providing a nucleation site for polymer deposition of the surface [81].

**Figure 6:**
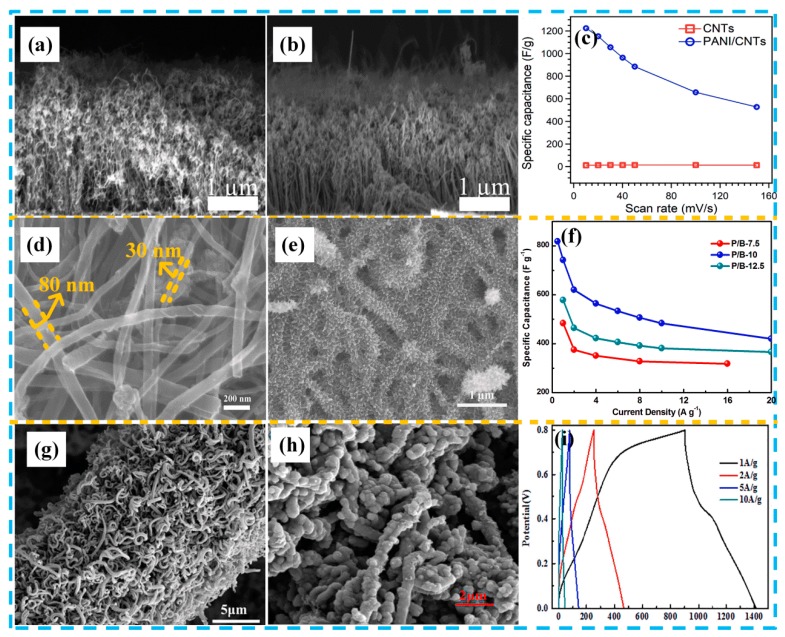
Morphology and electrochemistry of various PANI/CNT composites: (**a**) SEM image of CNTs; (**b**) SEM image of PANI/CNTs; (**c**) specific capacitance comparison of CNTs and PANI/CNTs at different scan rates. Reproduced with permission from Reference [82], copyright Elsevier, 2018. (**d**) SEM image of the buckypaper (multi-walled carbon nanotubes (MWCNTs) paper); (**e**) SEM image of the PANI nanothorns/buckypaper with the electrodeposition time of 10 min; (**f**) specific capacitance based on galvanostatic charge-discharge (GCD) measurements at different current densities pertaining to the PANI nanothorns/buckypaper. Reproduced with permission from Reference [83], copyright IOP science, 2019. (**g**) SEM images of the acidized carbon fibers (CF)-CNTs; (**h**) SEM images of the acidized CF-CNTs/PANI; (**i**) GCD curves at different current densities of the CF-CNTs/PANI electrode with polymerization time of 4 h. Reproduced with permission from Reference [84], copyright Elsevier, 2018.

Plasma polymerization is a green, fast, low cost, simple, and solvent free technology. It can synthesize ultra-thin polymer films with controlled thicknesses in the nanometer range. Plasma-based polymerization techniques are promising methods for fabricating PANI/CNT nanocomposites. Hussain et al. [82] used a radio frequency plasma-enhanced chemical vapor deposition method to synthesize vertically aligned CNTs on a TiN/SiO_2_/Si substrate using a Ni catalyst. As shown in Figure 6a, the prepared CNTs were vertically aligned (2 μm in length). This vertical array of CNTs ensures a uniform pore structure, good electron transport channels, and high ion diffusion rates. Subsequently, using the radio frequency-plasma polymerization method, the PANI film was uniformly covered on the surface of the CNTs, as shown in Figure 6b. The specific capacitances of vertically aligned CNTs and PANI/CNT electrodes (Figure 6c) reached 12 F g^−1^ and 1225 F g^−1^, respectively. The PANI/CNT electrodes showed a good cycling stability (65%, 15 A g^−1^, 5800 cycles) in a three-electrode system. Electrochemical in situ polymerization is another commonly used technique to direct polymer molecules to grow vertically along a skeleton and further form a three-dimensional (3D) structure. The vertical 3D PANI attached to the surface of the CNTs prevents self-aggregation, due to high surface energy, and effectively shortens the diffusion path and promotes the transport of electrons and ions. In our previous work, we synthesized new three-dimensional tapered PANI nanothorns on a buckypaper substrate (Figure 6d,e) by a simple electropolymerization process [83]. Benefiting from the synergistic effect of vertical growth of three-dimensional PANI and excellent electrical conductivity of MWCNTs, it can effectively alleviate the low-rate performance and cycle stability caused by the expansion and contraction of PANI in the long-term charge/discharge cycle of electrode materials, and provides a fast electronic transmission path. Finally, the composite electrode exhibited excellent high specific capacitance (742 F g^−1^ at 1 A g^−1^) and cycle stability (76% after 2000 cycles), as shown in Figure 6f. Luo et al. [84] prepared helical CNTs on the surface of CFs using chemical vapor deposition, and then combined it with PANI by in situ polymerization, as shown in Figure 6g,h. Flexible supercapacitor electrodes, based on spiral CNTs and PANI, exhibited high capacitance (660 F g^−1^ at 1 A g^−1^) and low interface charge transfer resistance (0.5 Ω). The all-solid helical CNTs/PANI flexible supercapacitor also exhibited good specific capacitance (439 F g^−1^ at 0.05 A g^−1^) and excellent deformation stability (95.4%, 500 bending/recovery cycles). This section also summarizes the electrochemical performance of typical PANI/CNT materials, as shown in Table 3.

### 2.4. Carbon Fibers/Polyaniline Hybrids

CFs are a versatile carbon matrix material with a carbon content of more than 90%. Most of the CFs are fibrous and consist of a carbon layer, consisting of graphite crystallites or flakes oriented along the fiber axis [85]. The main raw materials for the preparation of CFs are generally classified into polyacrylonitrile-based, asphalt-based and viscose-based preparations. “Carbon fiber” is actually a general term for a variety of CFs, because each type of product differs in raw materials, processes, and properties. CF materials include carbon nanofibers (CNFs), carbon cloth (CC), and carbon fiber paper (CFP). The preparation process of CFs includes stabilization treatment, carbonization, and graphitization. This preparation process endows CFs with excellent tensile strength, high modulus, high rigidity, excellent temperature resistance, fatigue resistance, and good electrical conductivity [86]. In the past few decades, CFs and CF composites have shown excellent market prospects in the fields of aerospace, building materials, automobiles, and biomedical instruments. In addition, CFs are usually selected as excellent three-dimensional skeletons or current collectors, due to their high flexibility, low weight, high electrical conductivity, corrosion resistance, and high temperature resistance.

**Figure 7:**
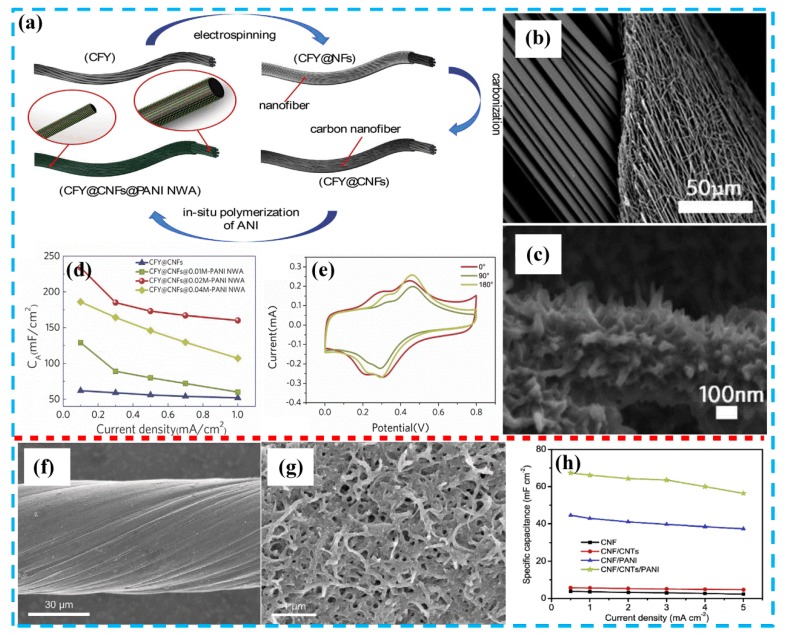
(**a**) Schematic diagram showing the hierarchical structure of CF yarn@carbon nanofiber (CNF) @PANI nanowire array; (**b**) SEM image of CF yarn@CNF; (**c**) SEM image of CF yarn@CNT@PANI nanowire array; (**d**) electrochemical performance at different current densities pertaining to different samples; (**e**) CV curves of different samples under different bending angles, with 0°, 90°, and 180° at scan rate of 20 mV s^−1^. Reproduced with permission from Reference [87], copyright Elsevier, 2018. (**f**) SEM image of pristine CNT fiber; (**g**) SEM image of CNF/CNT/PANI; (**h**) areal specific capacitances of CNF-, CNF/CNTs-, CNF/PANI-, CNF/CNT/PANI-supercapacitors at different current densities. Reproduced with permission from Reference [73], copyright Elsevier, 2018.

Benefiting from excellent electrical conductivity and flexibility, the application of CFs in new wearable electronic devices has attracted increasing attention. It is an effective way to improve the energy storage performance of the device by further loading the PANI on the CF substrate. In this section, we summarize some of the literature on PANI/CFs composites. Mao et al. [87] reported a method for synthesizing a sheath-core PANI nanowire array on the surface of aligned CNF/CF yarn. The precursor of CF yarn@CNF was prepared by an electrospinning process, using 10 wt% PAN and 90 wt% DMF as an electrospinning solution, as shown in Figure 7a. Subsequently, the precursor was carbonized to obtain CF yarn@CNF at 800 °C for 2 h under N_2_ atmosphere. The prepared CF yarn@CNF (Figure 7b) has high electrical conductivity and mechanical properties and is an excellent candidate for flexible electrodes. Finally, PANI (Figure 7c) was synthesized by a wet chemical method using the CFs as a matrix, and the energy storage performance was further improved. The assembled solid supercapacitor exhibited high specific capacitance (234 mF cm^−2^ at 0.1 mA cm^−2^), energy density (21.4 μWh cm^−2^), and power density (0.52 mW cm^−2^) and high cycle stability (90% after 8000 cycles) in the EMISBF_4_/PVDF/DMF gel electrolyte. Liu et al. [73] performed a beneficial modification of the CFs by depositing MWCNTs on the surface of CFs in 1mg mL^−1^ MWCNTs-COOH aqueous via electrophoretic deposition. A uniform PANI film was deposited on the CF surface using conventional electropolymerization. The assembled all-solid supercapacitor used phosphoric acid-polyvinyl alcohol gel electrolytes and exhibited high specific capacitance (67.31 mF cm^−2^ at the current density of 0.5 mA cm^−2^) and cycle stability (90% after 5000 cycles). In addition, it is worth mentioning that the composite material has good resistance to bending and deformation. Specifically, 99.8% of the capacitance can be maintained for 500 bends at 180°. Therefore, this simple, functional, and low cost CF/PANI composite provides a viable method for the production of high performance flexible supercapacitors.

## 3. Summary and Outlook

This review presents a state-of-the-art research direction and its limitations in supercapacitors for PANI/carbon hybrids. PANI is often used to prepare supercapacitor electrode materials, and high specific capacitance is its most significant advantage. It should not be neglected that the use of a conductive polymer as a supercapacitor electrode material may result in a rapid decrease in specific capacitance, due to the destruction of the polymer chain and poor cycle performance, due to volume expansion and contraction. Functional carbon materials have excellent physical and chemical properties, such as various porous structures, excellent specific surface area, good electrical conductivity, and accessibility to active sites, making them the best candidate framework materials for enhancing PANI performance. Overall, the different carbon morphologies from one-dimensional to three-dimensional structures endow carbon/PANI composites with better energy storage performance. Among these substrates, one-dimensional carbon materials represented by CNTs are considered to be ideal substrate materials for directional electron transport because of their orientation characteristics in a certain direction. Two-dimensional carbon materials represented by graphene have been widely developed for preparing high energy density devices, due to their high electrical conductivity, workability, and high specific surface area. Three-dimensional carbon materials represented by CC and CFP are widely used in functional and flexible supercapacitors, due to their foldability, stretchability, and flexibility. In recent years, research on PANI/carbon hybrids has made important developments in the field of supercapacitors, but the breakthrough of this composite material still faces several key issues, such as how to solve the shrinkage and expansion of PANI, improve the weak interfacial interaction between carbon and PANI, improve cycle stability of PANI materials, and optimize microstructure of composites.

In spite of these challenges, we believe that the next generation of PANI/carbon composites should meet the functional and energy storage standards of new supercapacitors as research progresses. Based on the above analysis, future research directions can be carried out in the following directions: (1) For AC/PANI, the interconnected porous structure should be designed to optimize electron transport and avoid collapse of porous microstructures through optimal structural design. (2) For graphene/PANI, graphene should be better dispersed in PANI through reasonable experimental design to avoid stacking PANI and graphene, thereby improving electrochemical performance and cycle stability. (3) For CNTs and CFs/PANI, its innate physical structural advantages should be reflected. Flexible and foldable functional supercapacitor devices that meet different requirements should be designed through optimal design. (4) Solving the problem of poor stability of carbon/PANI composites in organic supercapacitors at high current densities.

## Figures and Tables

**Table 1:** Electrochemical performance of various supercapacitors fabricated based on AC/PANI material.

Material	Specific Capacitance	Capacity Retention (Cycle Number)	Energy, Power Density /Wh kg^−1^, kW kg^−1^	Ref.
Porous carbon nanosheets/PANI	512 F g^−1^ at 1 A g^−1^	91% (3000)	22.3, 14	[26]
Enteromorpha prolifera-derived AC/PANI	622 F g^−1^ at 1 A g^−1^	87% (2000)	/	[27]
Hierarchical porous carbon/PANI	531 F g^−1^ at 0.5 A g^−1^	96% (10,000)	17.3, 1.06	[28]
Bamboo carbon/PANI	277 F g^−1^ at 0.5 A g^−1^	92% (1000)	47.5, 0.22	[29]
Wood-derived porous carbon/PANI	347 F g^−1^ at 2 A g^−1^	67% (2500)	44.4, 3.15	[30]
N-doped porous carbon/PANI fiber	755 F g^−1^ at 1 A g^−1^	91% (1000)	/	[31]
N‑self-doped carbon framework/PANI	373 F g^−1^ at 1 A g^−1^	80% (5000)	22.2, 7.31	[32]
AC/PANI	378 F g^−1^ at 1 A g^−1^	/	/	[33]
Cellulose-derived carbon/PANI	3.3 F cm^−2^ at 1 mA cm^−2^	83% (3000)	/	[34]
Cellulose-derived porous carbons/PANI	765 F g^−1^ at 1 A g^−1^	91% (5000)	/	[35]

**Table 2:** Capacitance comparison of PANI/graphene composites obtained from different experimental methods.

Material	Specific Capacitance	Cycle Number	Capacity Retention	Ref.
Hollow graphene/PANI spheres	546 F g^−1^ at 1 A g^−1^	/	/	[46]
Self-healing PANI/GO	757 F g^−1^ at 1 A g^−1^	9000	93%	[47]
PANI nanofibers/rGO	692 F g^−1^ at 1 A g^−1^	1000	83.3%	[48]
Graphene/PANI layers/PANI nanorods	578 F g^−1^ at 1 A g^−1^	10,000	93%	[49]
Flower-like PANI/graphene hybrid	1510 F g^−1^ at 1 A g^−1^	1500	89%	[50]
Phytic acid assisted graphene/PANI	865.6 F g^−1^ at 1 A g^−1^	1000	82%	[51]
Sulfonated PANI/GO	1107 F g^−1^ at 1 A g^−1^	5000	94%	[52]
3D bacterial cellulose/graphene/PANI	645 F g^−1^ at 1 A g^−1^	1000	82.2%	[53]
PANI/rGO	423 F g^−1^ at 0.8 A g^−1^	1000	75%	[54]
PANI/rGO/functionalized carbon cloth	0.47 F cm^−2^ at 0.5 mA cm^−2^	10,000	75.5%	[55]
PANI nanorod arrays/graphene	0.23 F cm^−2^ at 0.1 mA cm^−2^	8000	86.9%	[56]
N-doped graphene/PANI hydrogels	514.3 F g^−1^ at 1 A g^−1^	1000	87.1%	[57]
Holey N-doped rGO/PANI	746 F g^−1^ at 1 A g^−1^	2000	97%	[58]
3D rGO/self-suspended PANI	480 F g^−1^ at 1 A g^−1^	10,000	94.16%	[59]
Graphene carbon sphere/PANI/rGO	446 F g^−1^ at 5 mV s^−1^	5000	88.7%	[60]

**Table 3:** Capacitance comparison of PANI/CNTs composites obtained from different experimental methods.

Material	Specific Capacitance/F g^−1^	Capacity Retention (Cycle Number)	Ref.
3D PANI/CNT arrays	359 F g^−1^ at 1.56 mA cm^−2^	80.02% (5000)	[69]
PANI/GO/MWCNTs	696 F g^−1^ at 20 mV s^−1^	89% (3000)	[70]
PANI/CNTs/Graphene	0.79 F cm^−2^ at 1.5 mA cm^−2^	76% (3000)	[71]
RGO/CNTs/PANI	359 F g^−1^ at 1 A g^−1^	80.5% (2000)	[72]
CNF/CNTs/PANI	0.067 F cm^−2^ at 0.5 mA cm^−2^	90% (5000)	[73]
MWCNTs/PANI	809.6 F g^−1^ at 25 mV s^−1^	78% (2000)	[74]
CNT/PANI	0.016 F cm^−2^ at 0.25 mA cm^−2^	63% (500)	[75]
CNT/PANI network	632 F g^−1^ at 1 A g^−1^	/	[76]
CNF-CNTs/PANI	5.6 F cm^−2^ at 2 mA cm^−2^	85% (5000)	[77]
Polylactic acid/PANI/CNTs	510 F g^−1^ at 1 A g^−1^	99.5% (2000)	[78]
